# Design and radiosynthesis of class-IIa HDAC inhibitor with high molar activity via repositioning the ^18^F-radiolabel

**DOI:** 10.1038/s41598-024-65668-z

**Published:** 2024-07-02

**Authors:** Sulan Xu, Chun-Han Huang, Christopher Eyermann, Georgios V. Georgakis, Nashaat Turkman

**Affiliations:** 1 Stony Brook Cancer Center, Stony Brook, Long Island, NY 11794 USA; 2 Department of Radiology, School of Medicine, Stony Brook University, Stony Brook, NY 11794 USA; 3 Department of Biomedical Engineering, Stony Brook University, Stony Brook, NY 11794 USA; 4 Department of Surgery, School of Medicine, Stony Brook University, Stony Brook, NY 11794 USA

**Keywords:** Biochemistry, Drug discovery, Chemistry

## Abstract

The design and radiosynthesis of [^18^F]NT376, a high potency inhibitor of class-IIa histone deacetylases (HDAC) is reported. We utilized a three-step radiochemical approach that led to the radiosynthesis of [^18^F]NT376 in a good radiochemical yield, (17.0 ± 3%, decay corrected), high radiochemical purity (> 97%) and relatively high molar activity of 185.0 GBq/µmol (> 5.0 Ci/µmol). The repositioning of the ^18^F-radiolabel into a phenyl ring (^18^F-Fluoro-aryl) of the class-IIa HDAC inhibitor avoided the shortcomings of the direct radiolabeling of the 5-trifluoromethyl-1,2,4-oxadiazole moiety that was reported by us previously and was associated with low molar activity (0.74–1.51 GBq/µmol, 20–41 mCi/µmol). This radiochemical approach could find a wider application for radiolabeling similar molecules with good radiochemical yield and high molar activity.

## Introduction

**Figure 1:**
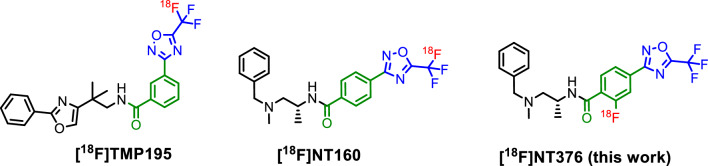
Chemical structures of the previously reported radiolabeled inhibitors of class-IIa HDACs^23,26^ and current work.

Class-IIa histone deacetylases (class-IIa HDACs) play a key role in various cancers^1–6^ and in the diseases of the central nervous system (CNS) such as Alzheimer’s and Huntington’s diseases^7–11^. Specifically, the dysregulation of class-IIa HDACs leads to memory and cognitive impairment, dementia and behavioral changes among others^12–20^. Therefore, we aimed to image the class-IIa HDACs expression in the CNS using brain penetrant positron emission tomography (PET) tracers. TMP195^21,22^ provided us with a good starting point to determine the feasibility of developing a brain penetrant class-IIa HDAC PET probe. We performed a structure–activity relationship (SAR) study using TMP195 as a lead inhibitor and we successfully identified new tracer candidates with IC_50_ in the low nM range for class-IIa HDAC4 and 5 (IC_50_ < 20 nM), and low potency (IC_50_ > 500 nM) to class-I/IIb HDAC isoforms^23^. The low potency to other HDAC classes is essential to eliminate off-target inhibition/imaging in the CNS since other HDAC classes are also known to be expressed in the brain^24,25^. Our medicinal chemistry campaign identified NT160 as a class-IIa HDAC inhibitor with superior potency (IC_50_: sub-to-low nM) for HDAC4 and 5 (Fig. 1)^23^. We then radiosynthesized [^18^F]NT160 using our novel late-stage incorporation of the ^18^F-fluoride into the 5-trifluoromethyl-1,2,4-oxadiazole (TFMO) moiety in acceptable radiochemical yield (3–5%) and high radiochemical purity (> 97%)^23,26^. However, the modest molar activity of [^18^F]NT160 (0.55–0.96 GBq/µmol, 15–26 mCi/µmol) is likely attributed to the isotopic dilution associated with ^18^F-CF_3_ labelling. The low molar activity is associated with high amount of nonradioactive mass dose which may hinder clinical PET imaging due to excessive self-blocking and unwanted pharmacological effect. Therefore, we explored repositioning the ^18^F-radiolabel into a different part of the molecule (i.e. phenyl ring) to produce our PET tracers with high molar activity (Fig. 1).

The high specificity and high selectivity of NT160 to class-IIa HDACs is attributed to the three distinctive pharmacophore motifs (Fig. 1): the cap moiety (in black) which interacts with the surface of the class-IIa HDAC enzyme, the linker moiety (in green) which occupies a hydrophobic channel, and the zinc binding moiety (in blue) that interacts with the zinc ion at the bottom of the catalytic pocket^23,27^. These motifs were the target for modification via structural activity relationship (SAR) studies to improve or maintain potency and selectivity to class-IIa HDACs.^23^ Therefore, we hypothesized that incorporating a fluorine substituent into the linker moiety is unlikely to alter the specificity and selectivity of NT376 to class-IIa HDACs. Indeed, our data below demonstrate the maintenance of high potency and selectivity of NT376 to class-IIa HDACs.

## Results

### Chemical synthesis of NT376

**Figure 2:**
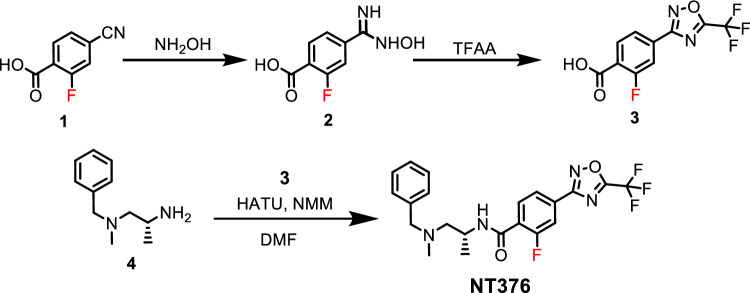
Chemical synthesis of NT376.

The chemical syntheses as shown in Fig. 2, started with the commercially available 4-cyano-2-fluorobenzoic acid (1) which was heated under reflux with hydroxylamine (NH_2_OH) to produce 2-fluoro-4-(N-hydroxycarbamimidoyl)benzoic acid (2) in quantitative yield. Compound 2 was then reacted with trifluoroacetic anhydride (TFAA) in pyridine to afford the key intermediate 2-fluoro-4-(5-(trifluoromethyl)-1,2,4-oxadiazol-3-yl)benzoic acid (3) in 70–80% yield. The key compound 3 was coupled with (*R*)-*N*^*1*^-benzyl-*N*^*1*^-methylpropane-1,2-diamine (4) in dimethylformamide (DMF) using 1-[bis(dimethylamino)methylene]-1*H*-1,2,3-triazolo[4,5-*b*]pyridinium 3-oxid hexafluorophosphate (HATU) and excess of 4-methylmorpholine (NMM) to afford the desired (*R*)-*N*-(1-(benzyl(methyl)amino)propan-2-yl)-2-fluoro-4-(5-(trifluoromethyl)-1,2,4-oxadiazol-3-yl)benzamide (NT376) in 65% yield.

### Biochemical evaluation of NT376

The next step was to determine the specificity, selectivity and potency of NT376 to class-IIa HDACs against endogenous class-IIa HDACs in HT-29 cells using the class-distinguishing substrates similar to our previous work^23^.

**Figure 3:**
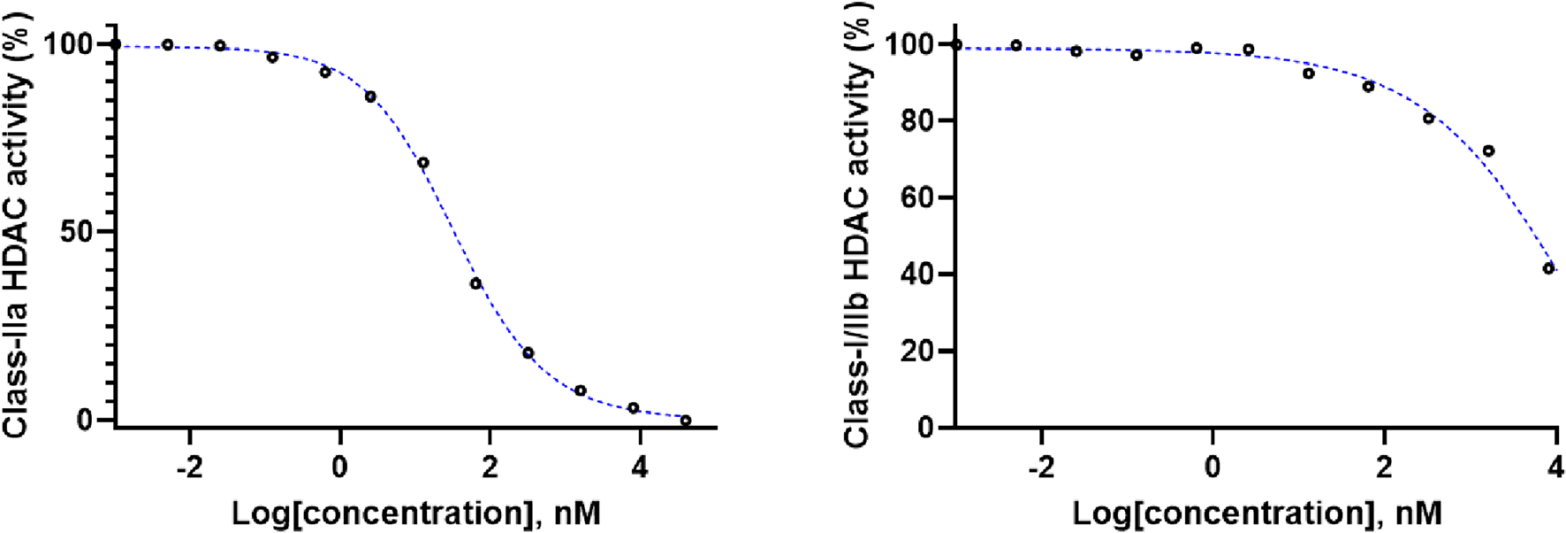
Selective endogenous class-IIa HDACs inhibition by NT376 in HT-29 cell line.

As shown in Fig. 3 and Table 1, NT376 exhibited high selectivity and high potency to class-IIa HDACs (cellular IC_50_ = 32 ± 7.6 nM) over other HDAC classes (cellular IC_50_ =  > 4.0 µM). The binding potency of NT376 to class-IIa HDACs was similar to that of NT160 (cellular IC_50_ = 46 ± 15 nM)^23^. Furthermore, the isoform specific assay (Table 1) further confirmed the high potency of NT376 to class-IIa HDAC4 and 5 isoforms and low potency to isoforms from other HDAC classes (HDAC1, class-I; HDAC6, class-IIb; and HDAC11, class-IV). Notably, NT376 performed slightly better than NT160 in the functional cell-based assay. On the other hand, NT160 was superior in the isoform-based assay. It is likely that these differences could be attributed to differential transportation across the cell membrane.

**Table 1:** IC_50_ values (nM ± SEM) for HDACs inhibition in HT-29 cells obtained using class-distinguishing fluorogenic substrates whole-cell assay.

	Whole-cell assay (HT-29 cells) IC_50_ (nM)		Isoform assay IC_50_ (nM)				
	Class IIa	Class I/IIb	HDAC4	HDAC5	HDAC1	HDAC6	HDAC11
NT160^23^	46 ± 15	> 5000	0.08 ± 0.02	1.2 ± 0.17	2383	> 10^5^	581
NT376	32 ± 7.6	> 4000	1.26	2.85	1483	5035	4851

The IC_50_ measurements were performed in triplicates and experiments were repeated twice.

### Chemical synthesis of precursors for radiochemistry

**Figure 4:**
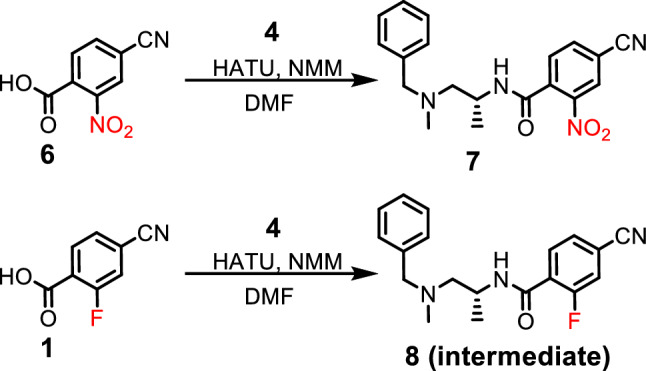
Synthesis of the key nitro-precursor **7** and intermediate **8**.

We designed a novel route to prepare key precursor-7 and its fluorinated analog 8 to modify the linker moiety in preparation for the radiochemical synthesis as shown in Fig. 4. The synthesis started with the preparation of the key nitro-precursor: R-*N*-(1-(benzyl(methyl)amino)propan-2-yl)-4-cyano-2-nitrobenzamide (7) which was synthesized by coupling the amine 4 and the 4-cyano-2-nitrobenzoic acid (6) using HATU and NMM, and similarly, the intermediate (*R*)-*N*-(1-(benzyl(methyl)amino)propan-2-yl)-4-cyano-2-fluorobenzamide (8) was synthesized.

### Radiosynthesis of [^18^F]NT376

**Figure 5:**
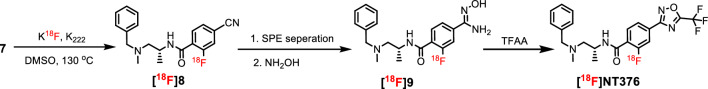
Radiosynthesis of [^18^F]NT376.

We designed a novel route to incorporate the ^18^F-radiolabel on the linker moiety as shown in Fig. 5. We utilized a multi-step approach to produce (*R*)-*N*-(1-(benzyl(methyl)amino)propan-2-yl)-2-[^18^F]fluoro-4-(5-(trifluoromethyl)-1,2,4-oxadiazol-3-yl)benzamide ([^18^F]NT376) as shown in Fig. 5. First, the key nitro-precursor 7 was reacted with potassium [^18^F]fluoride/kryptofix2.2.2 (K[^18^F]/K2.2.2) in dimethyl sulfoxide (DMSO) at 130 °C for 20 min to produce the key radioactive intermediate (*R*)-*N*-(1-(benzyl(methyl)amino)propan-2-yl)-4-cyano-2-[^18^F]fluorobenzamide ([^18^F]8). [^18^F]8 was trapped on a C18 SPE cartridge and was then eluted with methanol. [^18^F]8 was obtained in relatively high radiochemical purity (Fig. 4SI) and therefore, we were able to estimate the radiochemical yield to ~ 50% (crude, decay corrected, calculated based on the starting amount of radioactivity). The identity of the key intermediate [^18^F]8 was confirmed by co-injection together with the non-radioactive 8 into analytical radio-high-performance liquid chromatography (radio-HPLC) as shown in Fig. 4SI.

Afterwards, the methanol volume was reduced to ~ 0.2 mL and [^18^F]8 was treated with hydroxylamine solution (0.2 mL, 50% in water) and reacted at 100 °C for 10 min. The conversion of [^18^F]8 to [^18^F]9 was complete in ~ 10 min and the progress of the reaction was monitored by the disappearance of the analytical HPLC peak corresponding to [^18^F]8 and the appearance of new peak at the solvent front in line with a highly hydrophilic molecule (Fig. 4SI). Then, the solvent was evaporated to dryness and the residue was cooled in an ice-bath and reacted with trifluoroacetic anhydride (TFAA) for 10 min at 40 °C. The conversion of [^18^F]9 to [^18^F]NT376 was monitored and confirmed using radio-analytical HPLC.

Finally, [^18^F]NT376 was purified using a semi-preparative HPLC system (Fig. 6SI). The overall radiochemical yield of [^18^F]NT376 was 17 ± 3% (end of radiosynthesis, decay-corrected, n = 3), the radiochemical purity was > 97% and molar activity was 185.0 GBq/µmol (> 5.0 Ci/µmol). The radiochemical purity was determined from the chromatogram obtained from injection of [^18^F]NT376 into analytical HPLC (Fig. 7SI). The identity of [^18^F]NT376 was confirmed by co-injection together with the corresponding NT376 (unlabeled) using analytical HPLC, as shown in Fig. 8SI.

The molar activity of [^18^F]NT376 was determined from the area under the curve of the tracer corresponding to the ultraviolet peak in the HPLC chromatogram (Fig. 7SI) against a calibration curve pre-prepared with the unlabeled reference standard (Fig. 9SI). The molar activity of [^18^F]NT376 was > 185 GBq/µmol (> 5.0 Ci/µmol) which is significantly higher (> 190 fold) than that of [^18^F]NT160 (0.55–0.96 GBq/µmol, 15–26 mCi/µmol)^26^ and the previously reported ^18^F-trifluoromethyl-based tracers^28–30^.

## Discussion

The repositioning the ^18^F-radiolabel led to an efficient radiosynthesis of [^18^F]NT376 which was produced in relativity good radiochemical yield, high radiochemical purity and high molar activity. [^18^F]NT376 can be produced in a high radioactive dose (0.43 ± 0.1 GBq (11.5 ± 3 mCi)) and can be obtained when starting with ~ 5.55 GBq (~ 150.0 mCi), as needed for translational and future human studies. Furthermore, despite the multiple steps, the radiochemical synthesis is straightforward and can be accomplished in a relatively short period of time (~ 2 h) which will likely facilitate an automated radiosynthesis. Additionally, the molar activity is likely to further improve when starting with a high dose (currently we are starting with ~ 5.55 GBq (~ 150 mCi) and by performing the radiofluorination under automated settings.

There are several practical considerations to our current radiochemical approach. The nitro-precursor **7** can be easily prepared from the commercially available **4** and **6**. The radiosynthesis of [^18^F]8 is facilitated by the presence of the nitrile activating group. Moreover, the incorporation of the ^18^F-radiolabel into the aryl linker moiety may allow for facile generation of a library of radiolabeled class-IIa HDAC inhibitors by using the same radiosynthetic approach. In this regard, we are currently exploring the utility of this practical radiosynthetic approach to produce new radiolabeled class-IIa HDAC inhibitors by modifying the cap moiety (Fig. 1, moiety in black color). Lastly, the nitrile group is versatile and can be converted into tetrazole (via coupling/click with azides) or other functional groups such as amidine (Pinner reaction). We hypothesize that it may be potentially utilized to synthesize new scaffolds of radiolabeled imidazoles and tetrazines. Therefore, our radiochemical approach may find application in radiosynthesis of oxadiazoles, other heterocyclic and similar molecules^31–33^.

## Conclusions

In summary, we utilized an efficient radiofluorination approach that led to the radiosynthesis of TFMO containing molecules in good radiochemical yield (17.0 ± 3%), high radiochemical purity (> 97%) and high molar activity of 185.0 GBq/µmol (> 5.0 Ci/µmol). The repositioning of the ^18^F-radiolabel into the phenyl ring avoided the shortcomings of direct radiolabeling of the ^18^F-TFMO. Considering the versatility of the nitrile and the amide moieties, it is likely that our reported radiochemical approach can find a wider application in radiofluorination of similar target molecules.

### Methods

General information. Solvents and starting material were obtained from commercial sources and were used as received. High-performance liquid chromatography (HPLC) was performed with a 1260 series pump (Agilent Technologies, Stuttgart, Germany) with a built-in UV detector operated at 250 nm and a radioactivity detector with a single-channel analyzer (labLogic) using a semipreparative C18 reverse-phase column (10 × 250 mm, Phenomenex) and an analytical C18 column (4.6 × 250 mm, Ascentis RP-amide, Sigma). An acetonitrile/ammonium acetate buffer (MeCN/NH4OAc: 20 mM) with varying composition was developed specific to each compound. The quality control analyses were performed using analytical HPLC (Agilent Technologies, Stuttgart, Germany) at a flow of 1 mL/min. High resolution mass spectroscopy (HRMS) was performed using Agilent 1260HPLC/G6224A-TOF MS. NMR spectroscopy was performed using 400 MHz Bruker instrument.

#### Chemical synthesis

The chemical reactions and the biochemical evaluation of NT376 were performed analogous to methods described in our previous report^23,26,30^.

#### Biochemical evaluation of NT376.

We utilized the well-established fluorogenic assay to screen NT376 against recombinant human HDAC isoforms and against endogenous HDACs in HT-29 cells^23^. The class IIa HDAC fluorogenic assay functions in a two-step process: first, the class IIa HDAC removes the trifluoroacetate (TFA) from TFA-lysine substrate to generate free lysine which is recognized by trypsin and releases a fluorescent fluorophore (intact substrate is not recognized by trypsin) that can be detected using microplate reader. The IC_50_ was determined using nonlinear fit curves (GraphPad Prism) (% HDAC activity inhibition vs. compound concentration). The IC_50_ measurements were performed in triplicates and experiments were repeated twice with known HDAC inhibitors NT160 and SAHA used as positive and negative controls respectively.

Preparation of NT376: A 1.0 mM stock solution of NT376 was prepared in 100% DMSO. 11-dilutions of NT376 were then prepared in HDAC assay buffer with the DMSO adjusted to 2.5% final concentration in the assay medium.

#### Biochemical assay (HDAC isoform inhibition)

NT376 was screened against recombinant human HDACs isoforms using the commercially available HDACs enzyme assay kits (BPS Bioscience, Inc.). The experiments were performed according to the manufacturer protocol.

#### Cellular assay (endogenous HDAC inhibition)

The cell-based HDAC inhibitor assays were performed in HT-29 cells (ATCC). Briefly, cells were grown in DMEM media (Gibco) supplemented with 10% FBS (Thermo Fisher Scientific) and 1X Anti-Anti (Gibco) in a humidified incubator at 37 °C with 5% CO2. The day before the experiment, cells were washed with PBS and dissociated using 0.25% Trypsin–EDTA (Gibco). Trypsinization was stopped with media containing serum, and cells were collected by centrifugation. The cells were then washed with HDAC Assay Buffer (RPMI 1640 media without phenol red, Gibco, containing 0.1% FBS), re-collected by centrifugation, resuspended in HDAC Assay Buffer, and counted. HT-29 cells were plated into 96 well plates (200,000 cells/well) and placed into the incubator overnight. On the following day, the medium was aspirated from wells and NT376 (50 µl) was added to the wells followed by incubation for 3 h. Then the HT-29 cells were treated with either 100 µM Boc-Lys-TFA (class-IIa selective substrate) or 200 µM Boc-Lys-Ac (class-I/IIb selective substrate) in 45 µL cellular assay buffer (RPMI without phenol red, 0.1% Fetal Bovine Serum) and incubated for 3 h at 37 °C. The deacetylation was achieved by the addition of 50 µL HDAC developer solution (2.5 mg/ml trypsin in DMEM without Fetal Bovine Serum and 10% Tween 80), followed by incubation for one hour to sensitize the substrate and lyse the cells. Fluorescent counts were read with a microplate reader at an excitation wavelength of 360 nm and emitted light was detected at 460 nm.

#### Synthesis of 2-fluoro-4-(N-hydroxycarbamimidoyl)benzoic acid (2)

To the nitrile **1** (1.0 g, 6.0 µmol) in ethanol (30 mL) was added first hydroxylamine hydrochloric acid (1.0 g, excess) dissolved in water (8.0 mL) followed by sodium carbonate (1.5 g, excess) dissolved in water (12.0 mL). The mixture was heated under reflux for 4 h. Ethanol was removed under reduced pressure and the residue was diluted with water, acidified with 10% HCl to pH ∼3, and filtrated (Filter Funnel, Buchner, Medium Frit), then the white solid was washed with water (3 × 5.0 mL) and dried under reduced pressure at 70 ºC to afford compound **2** in 80% yield. Compound **2** was used in the following step without further purification. HRMS: Calculated for C_8_H_8_FN_2_O_3_ [M + H] 199.0513, Found: 199.0511.

#### Synthesis of 2-fluoro-4-(5-(trifluoromethyl)-1,2,4-oxadiazol-3-yl)benzoic acid (3)

Trifluoroacetic anhydride (2.0 mL) neat was added to compound **2** (0.8 g, 4.0 µmol) and the reaction mixture was heated to 50 °C under Argon for 3 h. The volatiles were removed by rotary evaporation and the crude product was purified by column chromatography using 5–10% methanol/dichloromethane. Compound** 3** was obtained as a white solid in quantitative yield. ^1^H NMR (CDCl_3_, 400 MHz) δ 8.22 (t, *J* = 8.0 Hz, 1H), 8.04 (d, *J* = 8.0 Hz, 1H), 7.99 (d, *J* = 8.0 Hz, 1H). ^19^F NMR (CDCl_3,_ 376.5 MHz) δ -65.26, 105.94. HRMS: Calculated for C_10_H_8_F_4_N_3_O_3_ [M + H]^+^ 294.0496, Found: 294.0496.

#### Synthesis of (R)-N-(1-(benzyl(methyl)amino)propan-2-yl)-2-fluoro-4-(5-(trifluoromethyl)-1,2,4-oxadiazol-3-yl)benzamide (NT376)

 The acid **3** (0.25 g, 0.91 µmol) and HATU (0.4 g, 1.10 µmol) in DMF (1.0–3.0 mL) were stirred for 15 min followed by simultaneous addition of the amine **4** (0.25 g, 1.4 µmol) and NMM (excess: ~ 1.0 mL). The reaction mixture was stirred for 3 h and then the DMF was removed by rotary evaporation (~ 70 ºC water bath). The residue was purified by column chromatography 60% ethyl acetate /hexane followed by trituration in cold pentane to afford the final products **NT376** in 63% yield. ^1^H NMR (CDCl_3,_ 400 MHz) δ 8.28 (t, *J* = 8.0 Hz, 1H), 8.06 (d, *J* = 8.0 Hz, 1H), 7.94 (d, *J* = 8.0 Hz, 1H), 7.32 (m, 5H), 7.01 (bs, 1H), 4.26 (m, 1H), 3.65 (d, *J* = 12.4 Hz, 1H), 3.52 (d, *J* = 12.4 Hz, 1H), 2.57 (t,* J* = 10.4 Hz 1H), 2.42 (dd, *J1* = 5.28 Hz, *J2* = 12.44 Hz 1H), 2.30 (s, 3H), 1.31 (d, *J* = 6.0 Hz, 3H). ^19^F NMR (CDCl_3,_ 376.5 MHz), δ -65.30, 111.56. ^13^C NMR (CDCl_3,_ 376.5 MHz), δ 167.73 (d, *J* = 2.5 Hz), 166.3 (q,* J* = 45.3 Hz), 161.96 (d, *J* = 2.5 Hz), 161.96 (d, *J* = 250 Hz, aryl-F), 138.8, 133.8 (d, *J* = 2.5 Hz),129.2 (d, *J* = 11.5 Hz), 128.9, 128.4, 127.2, 125.0 (d, *J* = 11.5 Hz), 123.8 (d, *J* = 3.6 Hz), 115.86 (q,* J* = 273.7 Hz, CF_3_), 115.6, 115.4, 62.7, 61.7, 44.5, 42.4, 19.0. HRMS: Calculated for C_21_H_21_F_4_N_4_O_2_ [M + H]^+^ 437.1595, Found: 437.1590. Chemical purity > 97% (HPLC).

Compounds **7** and **8** were synthesized analogous to NT376. The acids were coupled with the amine **4** using HATU and NMM.

#### Synthesis of (*R***)-***N*-(1-(benzyl(methyl)amino)propan-2-yl)-4-cyano-2-nitrobenzamide (7)

Compound **7** (key precursor) was synthesized in 55% yield. ^1^H NMR (CDCl_3,_ 400 MHz) δ 8.62 (s, 1H), 8.50 (d, *J* = 8.0 Hz, 1H), 8.48 (d, *J* = 8.0 Hz, 1H), 7.32 (m, 5H), 7.01 (bs, 1H), 4.19 (m, 1H), 3.63 (d, *J* = 12.0 Hz, 1H), 3.52 (d, *J* = 12.0 Hz, 1H), 2.65 (t,* J* = 9.40 Hz 1H), 2.47 (dd, *J1* = 5.28 Hz, *J2* = 12.44 Hz 1H), 2.28 (s, 3H), 1.32 (d, *J* = 6.4 Hz, 3H). HRMS: Calculated for C_19_H_21_N_4_O_3_ [M + H]^+^ 353.1608, Found: 353.1601.

#### Synthesis of (*R***)-***N*-(1-(benzyl(methyl)amino)propan-2-yl)-4-cyano-2-fluorobenzamide (8)

^1^H NMR (CDCl_3,_ 400 MHz) δ 8.22 (t, *J* = 8.0 Hz, 1H), 7.58 (d, *J* = 8.0 Hz, 1H), 7.48 (d, *J* = 8.0 Hz, 1H), 7.32 (m, 5H), 7.01 (bs, 1H), 4.21 (m, 1H), 3.63 (d, *J* = 12.0 Hz, 1H), 3.49 (d, *J* = 12.0 Hz, 1H), 2.54 (t,* J* = 9.40 Hz 1H), 2.44 (dd, *J1* = 5.28 Hz, *J2* = 12.44 Hz 1H), 2.30 (s, 3H), 1.30 (d, *J* = 6.4 Hz, 3H). ^19^F NMR (CDCl_3,_ 376.5 MHz), δ -110.85. HRMS: Calculated for C_19_H_21_FN_3_O [M + H]^+^ 326.1663, Found: 326.1658.

#### Radiochemistry

The preparation and the azeotropic drying of potassium [^18^F]fluoride/kryptofix2.2.2 was performed according to our previous reports^23,30^. Briefly, The [^18^F]F- was trapped on a QMA cartridge and then eluted with 80:20% acetonitrile/water solution (1.0 mL) containing kryptofix2.2.2 (12.0 mg) and K_2_CO_3_ (1.0 mg) to a V-vial (Wheaton) to produce potassium [18F]fluoride/kryptofix2.2.2. The water/acetonitrile mixture was removed under a stream of Argon at 110 °C. Then acetonitrile (3 × 1.0 mL) was added to the residue and the azeotropic drying was repeated under a stream of Argon at 110 °C.

#### Radiosynthesis of (*R*)-*N*-(1-(benzyl(methyl)amino)propan-2-yl)-4-cyano-2-[^18^**F]fluorobenzamide ([**^18^F]8)

A solution of the nitro-precursor (5.0 mg) in DMSO (0.4 mL) was added to the dried K[18F]/kryptofix2.2.2 and the mixture was heated at 130 ºC for 20 min. Water (3.0 mL) was added to the v-vial (reaction mixture) and the contents were transferred to another vial that contained water (15.0 mL). The mixture was passed through a C18 light SPE cartridge (Waters). The SPE cartridge was washed with water (2 × 10.0 mL) and the crude [^18^F]8 was eluted with methanol (0.4 mL). The identity of [^18^F]8 was confirmed by co-injection with the standard non-radioactive **8** (Fig. 4SI).

#### Radiosynthesis of (*R*)-*N*-(1-(benzyl(methyl)amino)propan-2-yl)-2-[18F] fluoro-4-(5-(trifluoromethyl)-1,2,4-oxadiazol-3-yl)benzamide ([^18^F]NT376)

The above **[**^**18**^**F]8** volume was reduced to ~ 0.2 mL under stream of argon while heating at 80 °C followed by the addition of hydroxylamine solution (0.2 mL, 50% in water) and reacted at 100 °C for 10 min. The conversion of **[**^**18**^**F]8** to** [**^**18**^**F]9** was complete in ~ 10.0 min and the progress of the reaction was monitored by the disappearance of the analytical HPLC peak corresponding to **[**^**18**^**F]8** and the appearance of new peak at the solvent front in line with a highly hydrophilic molecule (Fig. 5SI). Then, the reaction mixture was acidified by HCl (0.2 mL, 1.0 M) and solvent was evaporated to dryness at 100 °C with the aid of a stream of argon gas. The residue was cooled in an ice-bath and dichloromethane (0.2 mL) was added followed by trifluoroacetic anhydride (TFAA, ice cold). The reaction mixture was reacted for 10.0 min at 40 °C. The conversion of** [**^**18**^**F]9** to** [**^**18**^**F]NT376** was monitored and confirmed using analytical HPLC. The volatiles were evaporated to dryness at 40–60 °C with the aid of a stream of argon gas. The residue was dissolved in a solution (1.5 mL) containing 70% acetonitrile and 30% ammonium acetate (NH_4_OAc: 20 mM).

The purification of **[**^**18**^**F]NT376** was performed using semipreparative radio-HPLC system with a 1260 series pump (Agilent Technologies, Stuttgart, Germany) with a built-in UV detector operated at 250 nm and a radioactivity detector with a single-channel analyzer (labLogic). **[**^**18**^**F]NT376** was eluted from C18 reverse-phase column (00G-4041-N0: Luna® 5 µm, 100 Å, 250 × 10 mm, Phenomenex) using 68% acetonitrile and 32% ammonium acetate buffer (NH_4_OAc: 20 mM) at flow rate of 4.0 mL/minute. **[**^**18**^**F]NT376** was eluted and collected at 19–21 min post injection (Fig. 6SI). Water (20.0 mL) was added to the solution and the solution was trapped on a C18 light cartridge and eluted with ethanol (0.3 mL) and formulated for in vivo and in *vitro* studies. [^18^F]NT376 was formulated by the addition of a solution that contains 20% ethanol, 20% polysorbate 80 and 60% sodium ascorbate (5 mg/mL) solution.

Analytical HPLC was used for the quality control validation. The radioactive peak corresponding to **[**^**18**^**F]NT376** was detected with a radioactivity detector co-injected with the authentic non-radioactive compound **NT376** which was detected with ultraviolet detector (250 nm) (Fig. 8SI).

Generally, starting with ~ 5.55 GBq (~ 150 mCi) of [18F]F- led to a final dose of 0.43 ± 0.1 GBq (11.5 ± 3 mCi of **[**^**18**^**F]NT376** (on average, the radiosynthesis can be completed in two hours).

##### Molar activity

The molar activity was determined from the area under the ultraviolet peak curve corresponding to the non-radioactive trace in the analytical HPLC chromatogram (Fig. 7SI) against a calibration curve pre-prepared from the unlabeled reference standard (Fig. 9SI). The molar activity of **[**^**18**^**F]NT376** was > 185.0 GBq/µmol (> 5.0 Ci/µmol, n = 3) which is > 190 times higher than the 0.33–0.49 GBq/µmol (8.9–13.4 mCi/µmol) molar activity obtained for ^18^F-trifluoromethyl-containing tracers.^23,26,30^.

### Supplementary Information

Supplementary Information.

## Data Availability

All data generated or analyzed during this study are included in this published article [and its supplementary information files].
