# Supplementary material for: Design and radiosynthesis of class-IIa HDAC inhibitor with high molar activity via repositioning the 18F-radiolabel
Source: Sci Rep. 2024 Jul 2;14:15100. doi: 10.1038/s41598-024-65668-z (PMC11219833; doi:10.1038/s41598-024-65668-z)
Supplement: Supplementary file 1 — Supplementary Information. [file 41598_2024_65668_MOESM1_ESM.pdf]

Supporting Information

Design and radiosynthesis of a class-IIa HDAC inhibitor with high molar activity via repositioning the ^{18}F-radiolabel.

Sulan Xu[1,2], Chun-Han Huang[1,2,3], Christopher Eyermann[1,4], Georgios V. Georgakis[1,4], Nashaat Turkman*[1,2]

[1]Stony Brook Cancer Center, Stony Brook, Long Island, 11794, NY, USA

[2]Department of Radiology, School of Medicine, Stony Brook University, Long Island, 11794, NY, USA

[3]Department of Biomedical Engineering, Stony Brook University, Long Island, 11794, NY, USA

[4]Department of Surgery, School of Medicine, Stony Brook University, Long Island, 11794, NY, USA

Table of Contents **Page**

^{1}H NMR ($CDCl_3$, 400 MHz), B) ^{13}C NMR and C) ^{19}F NMR ($CDCl_{3,}$ 376.5 MHz) spectrums for NT376 2

High resolution spectroscopy for NT376 4

Analytical radio-HPLC chromatograms of [^{18}F]8 and [^{18}F]9 5

Semi-preparative high performance liquid chromatography (HPLC) purifiction of [^{18}F]NT376 5

Analytical radio-HPLC chromatograms of purified [^{18}F]NT376 co-injected with authentic NT376 6

Resferences 6

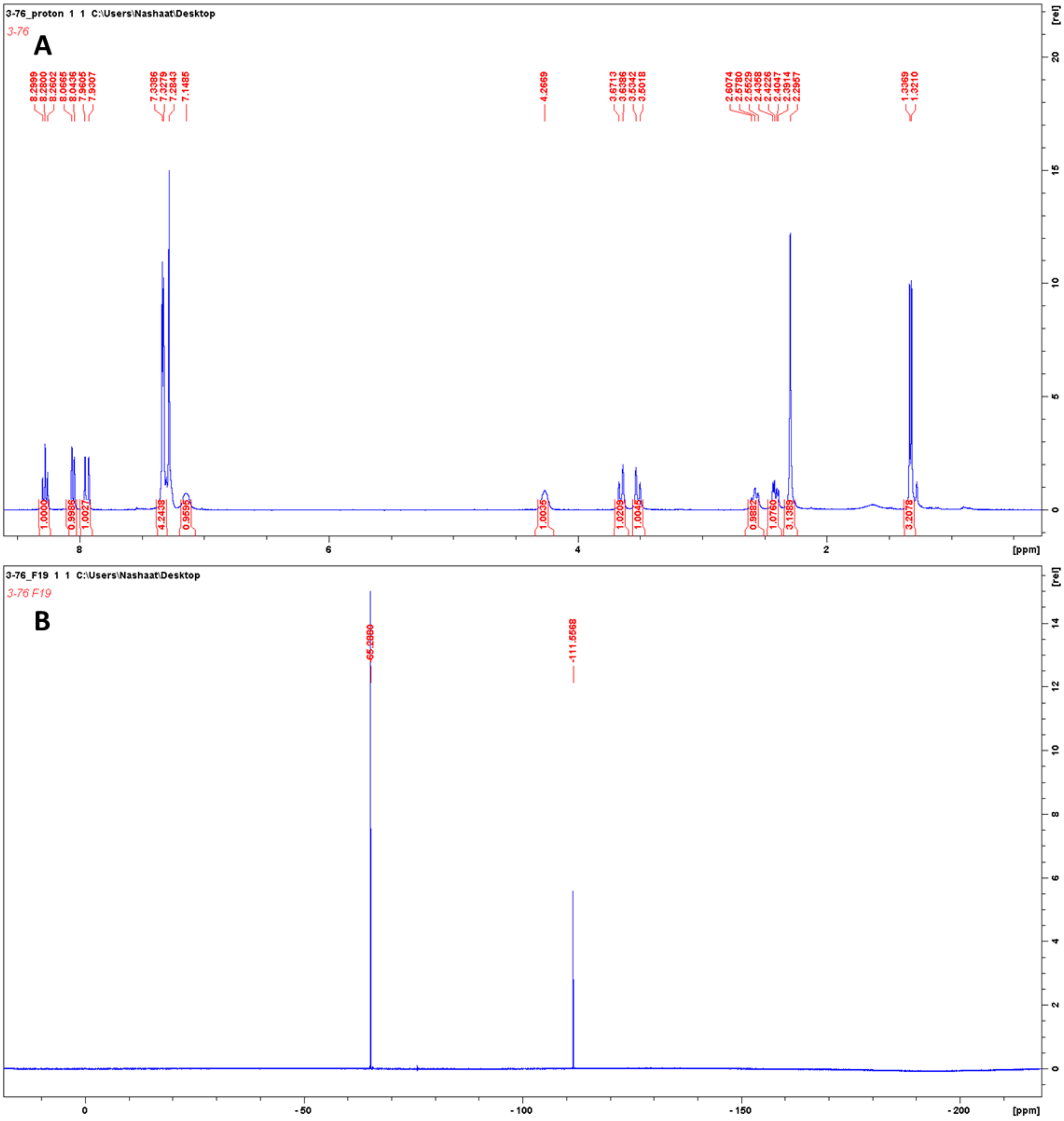

Figure 1. A) ^{1}H NMR ($CDCl_3$, 400 MHz) and B) ^{19}F NMR ($CDCl_3$, 376.5 MHz) spectroscopic characterization for NT376 was performed using 400 MHz Bruker instrument.

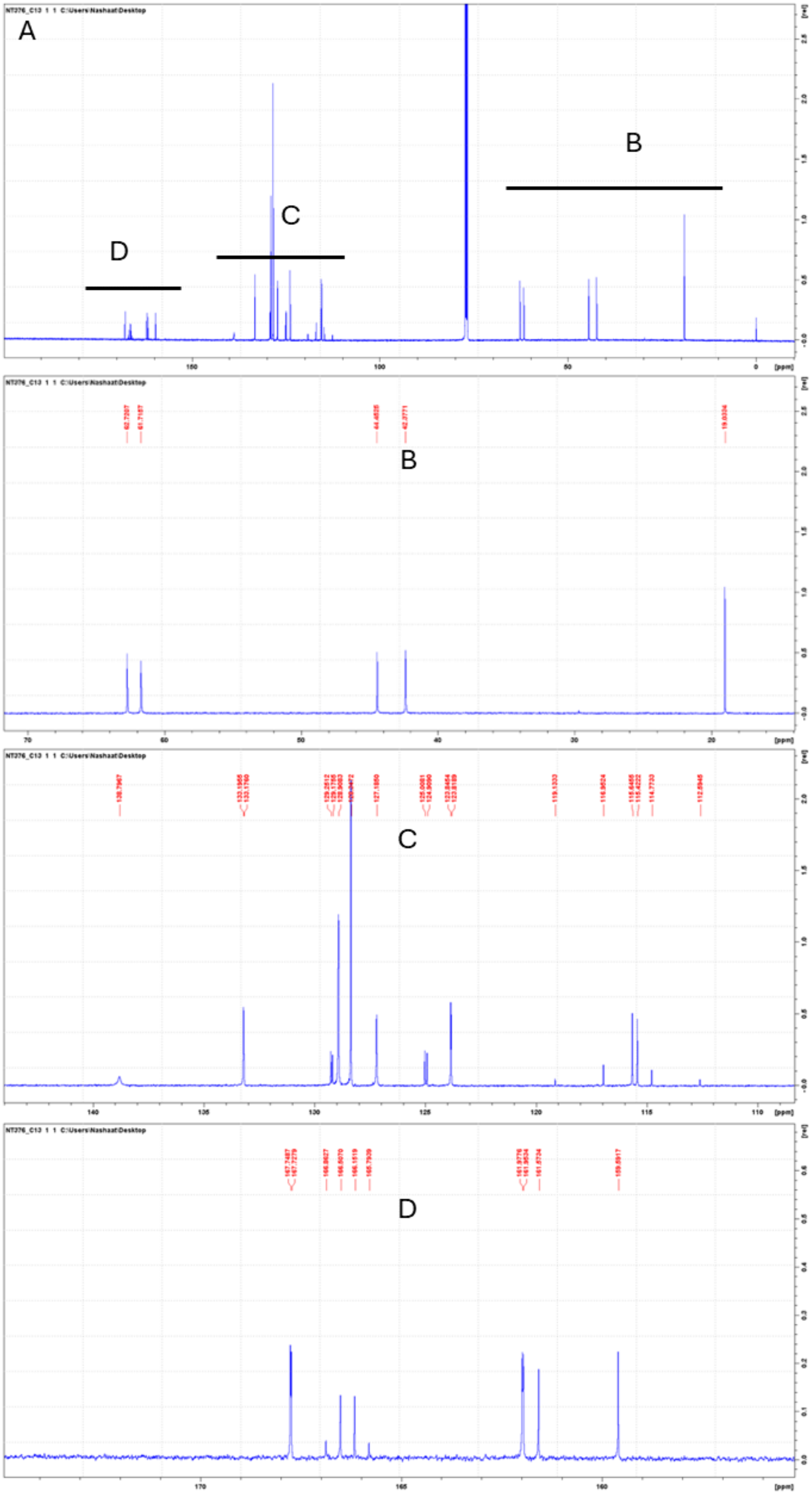

Figure 2. A) ^{13}C NMR, full spectrum for NT376 ($CDCl_3$, 400 MHz) and B-D: expanded spectrum of chemical shifts from up to down field (provided for clarity).

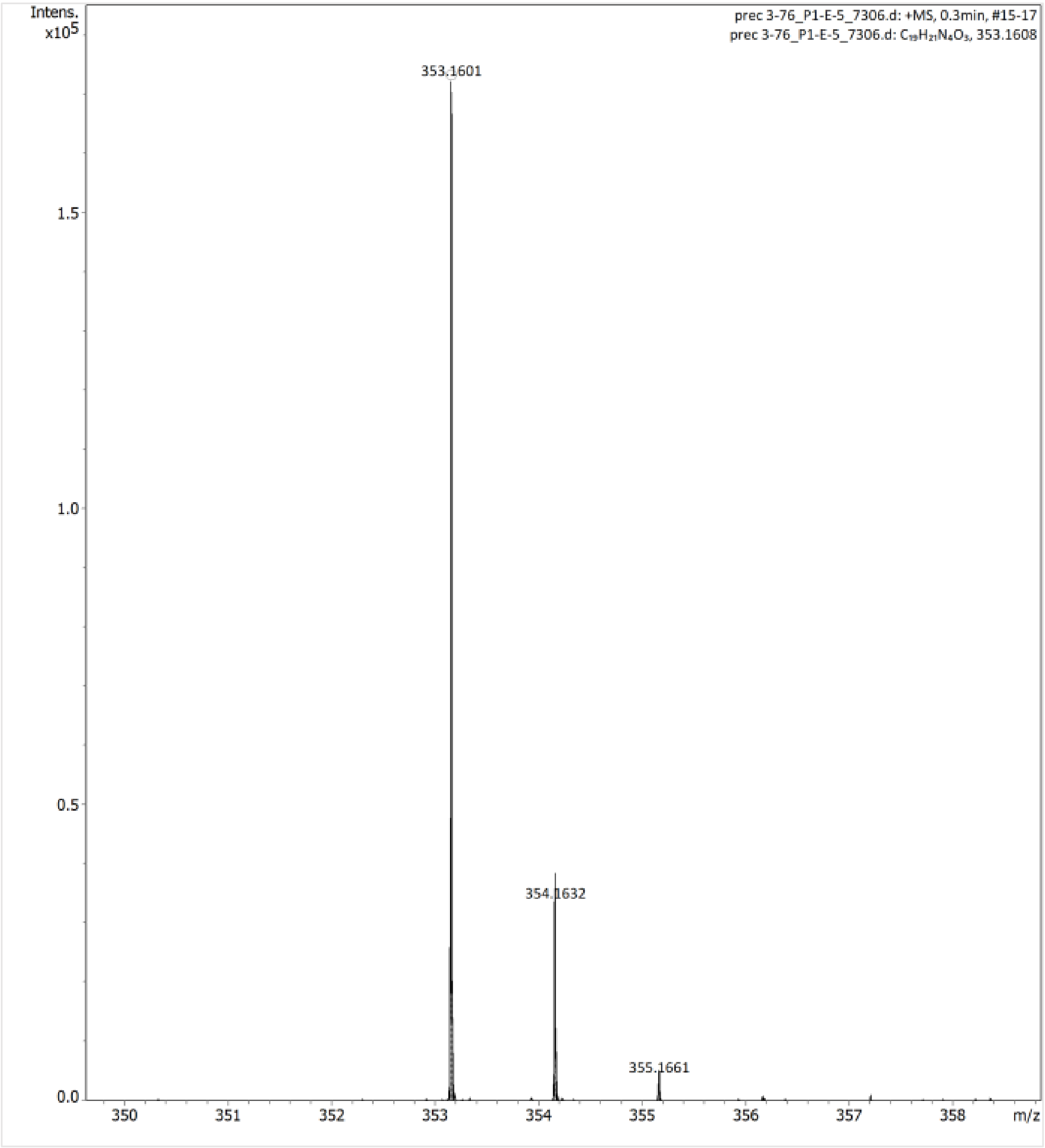

Figure 3. High resolution mass spectroscopy (HRMS) for NT376 was performed using Agilent 1260HPLC/G6224A-TOF MS.

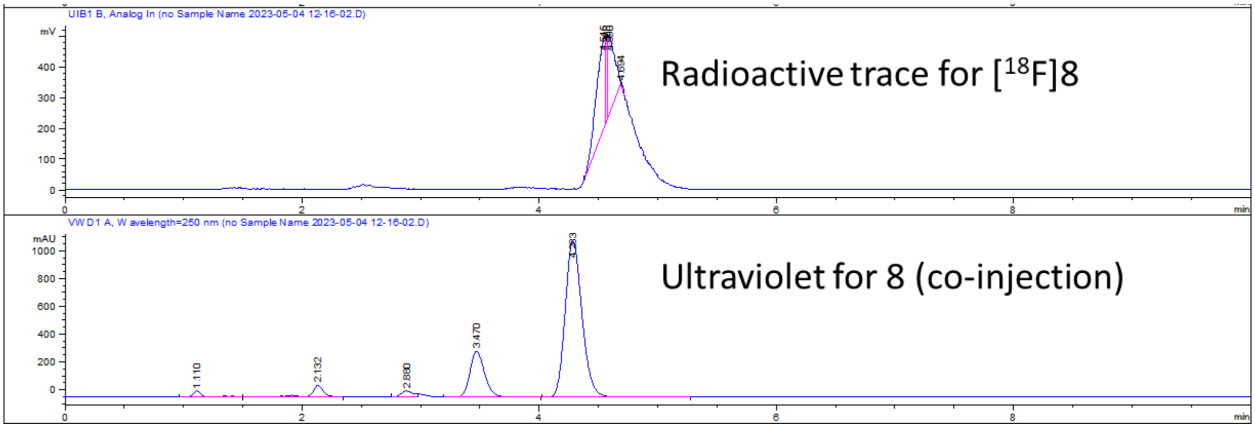

Figure 4. Analytical radio-HPLC chromatograms of [^{18}F]NT8 co-injected with authentic 8. Samples were eluted with 70% acetonitrile/30% ammonium acetate buffer (20.0 mM) at a flow rate of 1.0 mL/min.

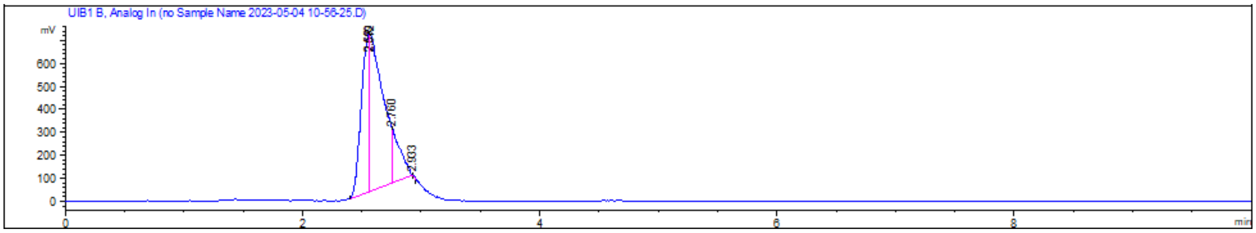

Figure 5. Analytical radio-HPLC chromatograms of [^{18}F]9 which demonstrate complete conversion of [^{18}F]8 to [^{18}F]9. Samples were eluted with 70% acetonitrile/30% ammonium acetate buffer (20.0 mM) at a flow rate of 1.0 mL/min.

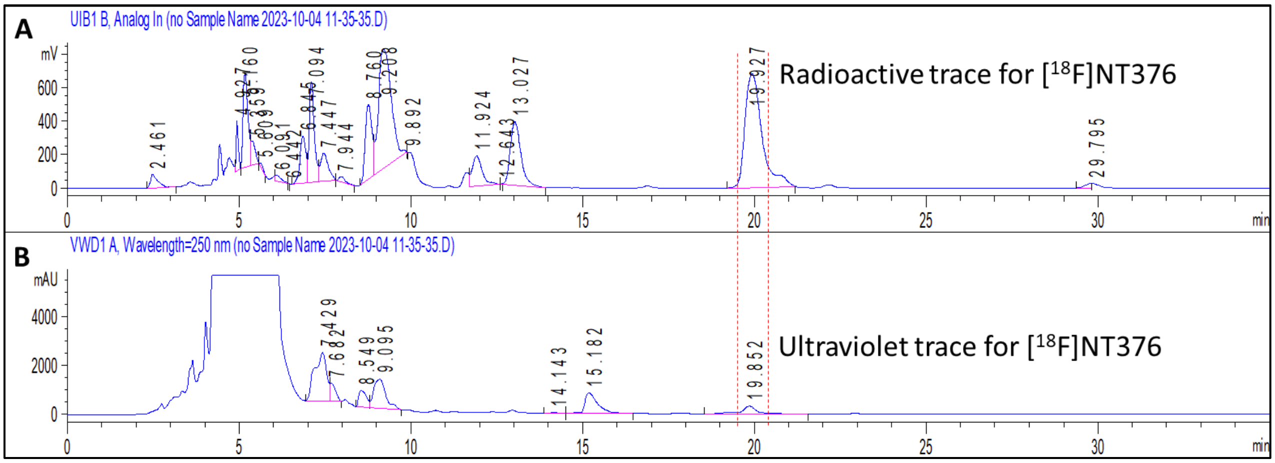

Figure 6. Semi-preparative high performance liquid chromatography (HPLC) purification of [18F]NT376 (a) radioactive peak of [^{18}F]NT376 in crude mixture detected with radioactivity detector, (b) [^{18}F]NT376 associated non-radioactive mass in the crude reaction mixture were detected with ultraviolet detector. Dashed lines shows start and stop of [^{18}F]NT376 collection which demonstrate significant improvement in molar activity compared to our previous reports.[1-3]

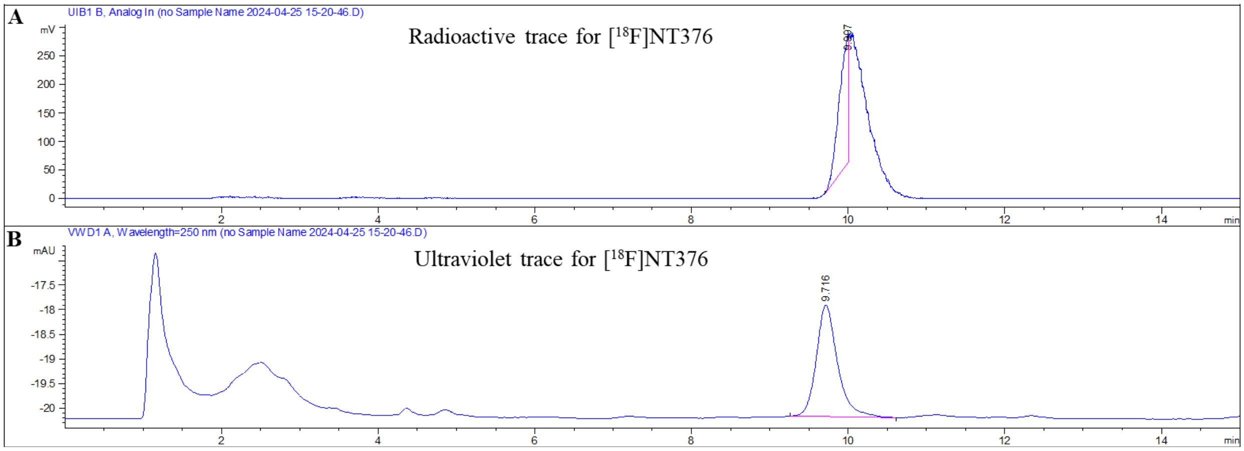

Figure 7. Analytical radio-HPLC chromatograms of purified [^{18}F]NT376: A) radioactive trace and B) ultraviolet mass trace for NT376 (note the Y-scale). Samples were eluted with 70% acetonitrile/30% ammonium acetate buffer (20.0 mM) at a flow rate of 1.0 mL/min.

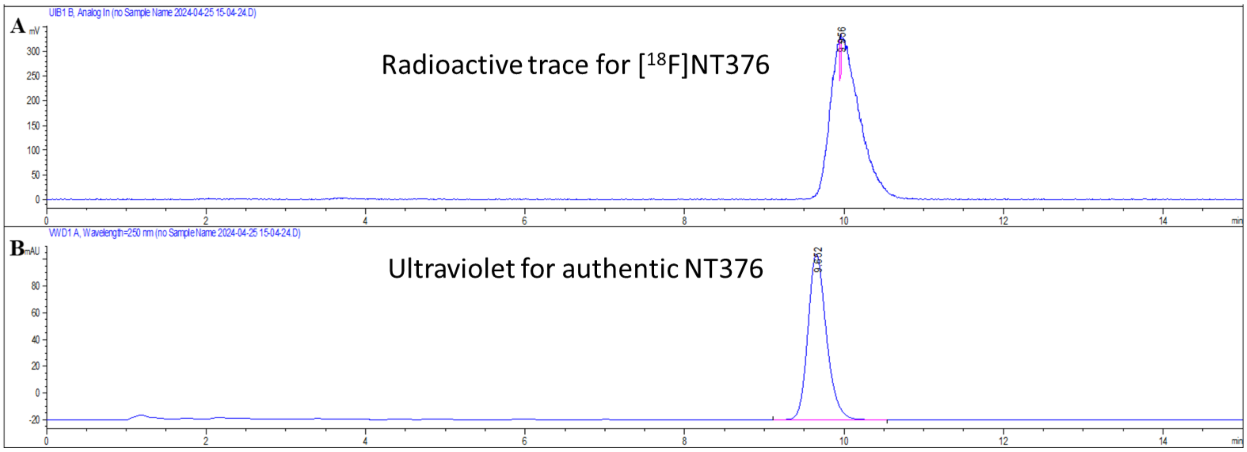

Figure 8. Analytical radio-HPLC chromatograms of quality control for [^{18}F]NT376: A) radioactive trace and B) co-injection with authentic NT376. Samples were eluted with 70% acetonitrile/30% ammonium acetate buffer (20.0 mM) at a flow rate of 1.0 mL/min.

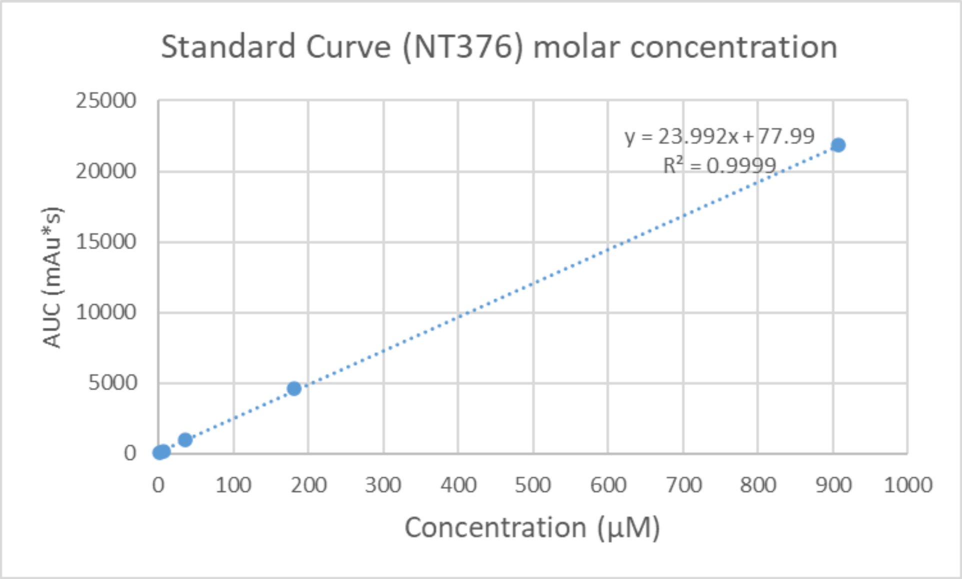

Figure 9. Standard curve obtained for NT376 and was used to determine the molar activity.

(1) Turkman, N.; Xu, S.; Huang, C. H.; Eyermann, C.; Salino, J.; Khan, P. High-Contrast PET Imaging with [(18)F]NT160, a Class-IIa Histone Deacetylase Probe for In Vivo Imaging of Epigenetic Machinery in the Central Nervous System. *J Med Chem* **2023**, *66* (8), 5611-5621. DOI: 10.1021/acs.jmedchem.2c02064.

(2) Turkman, N.; Liu, D.; Pirola, I. Design, synthesis, biochemical evaluation, radiolabeling and in vivo imaging with high affinity class-IIa histone deacetylase inhibitor for molecular imaging and targeted therapy. *Eur J Med Chem* **2022**, *228*, 114011. DOI: 10.1016/j.ejmech.2021.114011.

(3) Turkman, N.; Liu, D.; Pirola, I. Novel late-stage radiosynthesis of 5-[18F]-trifluoromethyl-1,2,4-oxadiazole (TFMO) containing molecules for PET imaging. *Sci Rep* **2021**, *11* (1), 10668. DOI: 10.1038/s41598-021-90069-x.
